# A variable sampling plan based on the coefficient of variation for lots resubmission

**DOI:** 10.1038/s41598-023-50498-2

**Published:** 2023-12-27

**Authors:** Ching- Ho Yen, Muhammad Aslam, Chia-Hao Chang, Rehan Ahmad Khan Sherwani, Liaquat Ahmad, Chi-Hyuck Jun

**Affiliations:** 1 Department of Technology for Smart Living, Huafan University, Taipei, Taiwan; 2 Department of Statistics, Faculty of Science, King Abdulaziz University, 21551 Jeddah, Saudi Arabia; 3 Department of Nursing, Department of Nursing, Chang Gung University of Science and Technology, Chiayi Campus, Chiayi, Taiwan; 4 College of Statistical Sciences, University of the Punjab, Lahore, Pakistan; 5 Department of Statistics and Computer Science, University of Veterinary and Animal Sciences, Lahore, Pakistan; 6 Department of Industrial and Management Engineering, POSTECH, Pohang, 790-784 Republic of Korea

**Keywords:** Engineering, Mathematics and computing

## Abstract

This study focuses on the issue of lots resubmission in inspection processes, which often arises when the initial inspection of a lot is suspected, marked as held, or not accepted. To address this problem, a novel variables sampling plan based on the coefficient of variation is proposed. The objective is to determine the sampling plan parameters that minimize the average sample number while satisfying the two-points of operating characteristic curve. Practical considerations are taken into account by providing tabulated values for the inspection sample size and acceptance criteria of the proposed plan. These tables incorporate various combinations of quality levels, considering commonly used producer's risk and consumer's risk. Furthermore, a comparative analysis between the proposed plan and a single sampling plan is conducted to highlight the advantages of the new approach. To illustrate the practical implementation of the proposed plan, an example is presented.

## Introduction

The acceptance sampling plan is an important tool used in quality control for the disposition of the manufactured lots or a sequence of lots, which aims to decide the acceptance or rejection of one lot instead of determining the quality of one lot^1^. The acceptance sampling plan gives the required sample size and judgment standard for lot disposition while reaching an agreement regarding the specific quality levels and risks between the producer and the purchaser. As the disposition of the lot is based upon the sampling theory, two types of errors are unavoidable, called Type-I error and Type-II error. Type-I error is the producer’s risk resulting in the rejection of good lots and is denoted as $\alpha$-risk. On the other hand, Type-II error is the purchaser’s risk resulting in the acceptance of bad lots and also denoted as $\beta$-risk. For both the two parties, the producer wishes to lower the risk of rejecting a good lot while the purchaser desires to lower the risk of accepting a bad lot. Therefore, a well-designed sampling plan should be able to effectively reduce the gap between the required quality level and the actual quality level supplied. For this purpose, the design of an acceptance sampling plan is usually based on the two-points of the operating characteristic curve (OC curve).

Depending on the property of quality characteristics, an acceptance sampling plan can be divided into two types, attributes sampling plan and variables sampling plan. An attributes sampling plan is used for quality characteristics expressed on a “go, no-go” basis while a variable sampling plan is used for quality characteristics measured as a numerical value. Compared with the attributes sampling plan, a variable sampling plan provides a smaller sample size to attain the same protection for producer and purchaser as well as giving more information about lots. Consequently, variable sampling plan attracts more and more attention from industries today. However, the traditional variable sampling plan does not cope with the lot sentencing effectively when the proportion defective in the process is very low. To overcome the defect, many authors have proposed variable sampling plans based on process capability indices (PCIs) for lot inspection. Pearn and Wu used the one-sided process capability indices (*C*_*pu*_ and *C*_*pl*_) and *C*_*pm*_ to design the single sampling plan, respectively^2,3^. Pearn and Wu developed a single sampling plan based on C_pk_^4^. Wu and Pearn proposed a single sampling plan based on C_pmk_^5^. Yen and Chang used L_e_ to develop a single sampling plan^6^. By extending the research^6^, Aslam et al. developed the repetitive sampling plan based on L_e_^7^and proposed the two-stage sampling plan based on L_e_^8^. Aslam et al. designed the repetitive group sampling plan based on *C*_*pk*_^9^. Aslam et al. developed the multiple dependent state repetitive sampling plan with process loss function L_e_^10^. Yen et al. firstly proposed the exponential weighted moving average (EWMA) method to develop the sampling plan based on the process yield index *S*_*pk*_^11^. Wu and Liu used the yield index *S*_*pk*_ to design the single sampling plan^12^. Aslam et al. used the multiple dependent state method to build the sampling plan based on L_e_ which considers the qualities of the current lot and preceding lots^13^. Wu et al. also used multiple dependent state method to develop the sampling plan based on *C*_*pk*_^14^. Yen et al. used the one-sided process capability indices (*C*_*pu*_ and *C*_*pl*_) to develop the repetitive sampling plan^15^. More information about PCI-based sampling can be seen in three references^12,16,17^.

There are three kinds of sampling plans that may have different numbers of sampling for lot sentencing, including the single, double, and multiple sampling plans. In a single sampling plan, the information obtained from one single sample is used to make a decision of accepting or rejecting the lot. Double sampling provides an extra opportunity to make a decision of acceptance or rejection of the lot if the decision regarding the lot could not be reached based on the information from the first sample. The multiple sampling plan is just an extension of the double sampling plan. Among them, the single sampling plan is most popularly used due to its simplicity in management. In most situations, we would decide to accept or reject the lot based on the information obtained from the single sample. However, resubmitting lots may occur after examination by the producer once a lot is not accepted based on a single sample. For example, the producer may argue the results of the first sample so that the same number of units resampled may be implemented under the regulations of the contract or statues^18^. In addition, to sustain the good partnership between the vendor and buyer, the resampling is often permitted for lots non- accepted on the original inspection. As mentioned, in certain countries, tax on products is assessed based on a sample result and if the producer does not agree with the result, a second sample result will be used^18^. Govindaraju and Ganesalingam developed a method of an attributes sampling plan for resubmitted lots which examined the situation where resampling is permitted for lots not accepted on original inspection^18^. For the issue of resubmitted lots, some researchers have used quality induces to develop the resubmitted sampling plans for lot sentencing^19–25^.

In specific cases, we may be more concerned with the stability of products so that the existing resubmitted sampling plans cannot be applied adequately. Take the tensile strength of steel, for example, the stability of tensile strength may be more important than the average tensile strength for enterprises who construct the buildings since the superstructure would be propped up well if the tensile strength of steel used is consistent. In addition, there are some situations in which the targeted quality characteristics have different units of measurement, which lead to the difficulty of comparison for such quality characteristics. For addressing such a situation, researchers used the relatively dimensionless measure *CV*, defined as the ratio of the standard deviation to the mean, to compare the different magnitudes of data sets. During recent years, the *CV* has attracted many researchers to engage in research on quality control^26–32^. By exploring the literature and best of the authors’ knowledge, the resubmitted sampling plans based on a *CV* are not proposed yet. Therefore, we attempt to develop a variables sampling plan based on a *CV* when resampling is permitted for lots non-accepted on the original inspection. The rest of the paper is organized as follows: a resubmitted sampling plan based on the *CV* is provided in “A sampling plan based on the CV for resubmitted lots” section. In “Determination of plan parameters” section the determination of the plan parameters is described. Discussion and analysis are made in “Discussion and analysis” section. In “An application example in industry” section, a practical example is presented to illustrate the proposed methodology. Finally, conclusions and recommendations are given in the last section.

## A sampling plan based on the *CV* for resubmitted lots

### Coefficient of variation

The coefficient of variation is a dimensionless number, which considers the spread of data relative to the central location. It is usually used to measure the consistency of data sets with different units or widely different means. Due to the properties of this index, it has been widely applied in many practical applications of quality control, such as reliability, control chart, acceptance sampling plan, and so on. *CV* is the ratio of the standard deviation to the mean, defined as

$$ CV = \frac{\sigma }{\mu } $$

where $\mu$ is the mean, and $\sigma$ is the standard deviation. In reality, the parameter *CV* is almost unknown so we have to use the sample statistic to estimate it. In order to estimate the *CV*, we consider the following natural estimator

$$ \mathop {CV}\limits^{ \wedge } = \frac{S}{{\overline{X}}} $$

where $\overline{X}$ is the sample mean, and $S$ is the sample standard deviation. Given the assumption that the data follows the normal distribution with mean $\mu$ and standard deviation $\sigma$, Iglewicz et al. showed that the statistic $\sqrt n /\mathop {CV}\limits^{ \wedge }$ is distributed as a non-central $t$ distribution with *n*-1 degrees of freedom and a non-central parameter $\sqrt n /CV$, denoted as $t_{n - 1,\;\sqrt n /CV}$^33^.

### The proposed methodology

Govindaraju and Ganesalingam^18^ first developed an attribute sampling plan for resubmitted lots, which permits the resampling to be executed for lots not accepted on the original inspection. Referring to this methodology, we propose a resubmitted sampling plan based on *CV*, whose operation procedure is stated as follows:

*Step 1*: Take a random sample of size $n$ from the lot and compute the estimated value of *CV*, $\mathop {CV}\limits^{ \wedge }$.

*Step 2*: Accept the lot if $\mathop {CV}\limits^{ \wedge } < k$, where *k* is the acceptance value under-sampling inspection. Otherwise, resubmit the lot and go to Step 1.

*Step 3*: On non-acceptance in Step 2, apply the plan *m* times and reject the lot if it is not accepted on (*m* − 1)st resubmission.

As^18^ stated, the eventual acceptance probability of resubmitted sampling plan can be expressed as

1 $$ P_{A} (p) = 1 - \left[ {1 - P_{a} (p)} \right]^{m} $$

where $P_{a} (p)$ is the probability of accepting a lot with proportion defective *p* for a single inspection. Also, the *ASN* with proportion defective *p* is written as

2 $$ \;ASN(p) = \frac{{n\left[ {1 - \left( {1 - P_{a} (p)} \right)^{m} } \right]}}{{P_{a} (p)}} $$

Referring to Eq. (1)-(2), the eventual acceptance probability and *ASN* of the resubmitted sampling plan based on a *CV* can be written respectively as

3 $$ P_{A} (CV) = 1 - \left[ {1 - P_{a} (CV)} \right]^{m} $$

and

4 $$ \;ASN(CV) = \frac{{n\left[ {1 - \left( {1 - P_{a} (CV)} \right)^{m} } \right]}}{{P_{a} (CV)}} $$

where $P_{a} (CV)$ is the probability of accepting a lot with a coefficient of variation *CV* for a single inspection, which can be expressed as

5 $$ P_{a} (CV) = P(\mathop {CV}\limits^{ \wedge } < k) = P\left( {t_{n - 1,\;\sqrt n /CV} > \sqrt n /k} \right) $$

Commonly, an acceptance sampling plan is designed based on the principle of two points on the OC curve. By minimizing the *ASN* while satisfying the two designated points ($CV_{AQL}$,$1 - \alpha$) and ($CV_{LTPD}$,$\beta$), the resubmitted sampling plan based on a *CV* can be built as

6 $$ Min\;\;\frac{{n\left[ {1 - P\left( {t_{{n - 1,\;\;2\sqrt n /(CV_{AQL} + CV_{LTPD} )}} < \sqrt n /k} \right)^{m} } \right]}}{{P\left( {t_{{n - 1,\;\;2\sqrt n /(CV_{AQL} + CV_{LTPD} )}} > \sqrt n /k} \right)}} $$

Subject to

7 $$ P_{A} (CV_{AQL} ) = 1 - P\left( {t_{{n - 1,\;\;\sqrt n /CV_{AQL} }} < \sqrt n /k} \right)^{m} \ge 1 - \alpha $$

8 $$ P_{A} (CV_{LTPD} ) = 1 - P\left( {t_{{n - 1,\;\;\sqrt n /CV_{LTPD} }} < \sqrt n /k} \right)^{m} \le \beta $$

where *CV*_*AQL*_ and *CV*_*LTPD*_ are acceptable quality level and lot tolerance percent defective for *CV* respectively, and $\alpha$ and $\beta$ are the producer’s risk and buyer’s risk respectively.

## Determination of plan parameters

For practical purpose, we provide the corresponding sample size required and acceptance value for the resubmitted sampling plan, with commonly used producer’s risk, buyer’s risk, and quality levels of coefficient of variation. Using the above-mentioned Eqs. (3)–(8), we write the SAS-Language code program to find the parameters of the proposed plan for different levels of risks and quality levels. Tables 1, 2, 3, 4 display (*n*,* k*, *ASN*) values for ($\alpha$,$\beta$) = (0.05, 0.1), (0.1, 0.05), (0.05, 0.05) and (0.1, 0.1), with some quality levels of ${{\text{L}}}_{{\text{AQL}}}$ $CV_{AQL}$ and ${{\text{L}}}_{{\text{LTPD}}}$ $CV_{LTPD}$. Referring to the parameters of the proposed plan in Tables 1, 2, 3, 4, the practitioners can determine the number of production items to be sampled and the corresponding critical values. For example, if the number of resampling permitted is 1, the benchmarking quality level (${{\text{L}}}_{{\text{AQL}}}$ $CV_{AQL}$, ${{\text{L}}}_{{\text{LTPD}}}$ $CV_{LTPD}$) is set to (0.06, 0.08) with producer’s $\alpha$-risk = 0.05 and buyer’s $\beta$-risk = 0.10, then the corresponding sample size, critical value, and *ASN* for the resubmitted sampling plan are 40, 0.0649 and 68.67, respectively. This implies that the lot will be accepted if the 40 items yield measurements with $\mathop {CV}\limits^{ \wedge } < {0}{\text{.0649}}$. Otherwise, resubmission is allowed once if the lot is not accepted on original inspection and the lot will be rejected if the resubmission is also not accepted. On the other hand, if we permit the number of resampling to be 2 given all other conditions are the same as the above, then the corresponding sample size and critical value for the resubmitted sampling plan are 34, 0.0619 and 83.72, respectively. This implies the consumer allows resubmissions twice in the case of non-acceptance on the submitted sample of size 34, and reject the lot if two resubmissions with the same sample size are not accepted.

**Table 1:** The parameters of the proposed plan for risks, $\alpha$ = 0.05 and $\beta$ = 0.1.

$CV_{AQL}$	$CV_{LTPD}$	*n*	*m* = 2		*n*	*m* = 3	
			*k*	*ASN*		*k*	*ASN*
0.05	0.06	94	0.0527	159.78	79	0.0512	191.35
	0.07	30	0.0547	51.7	26	0.0519	64.14
	0.08	17	0.0565	29.53	15	0.0524	37.55
	0.09	12	0.0581	21	11	0.0534	27.69
	0.10	9	0.0585	15.94	9	0.0548	22.7
0.06	0.07	130	0.0628	220.03	109	0.0612	264.16
	0.08	40	0.0649	68.67	34	0.0619	83.72
	0.09	22	0.0669	38.01	19	0.0625	47.33
	0.10	15	0.0685	26.13	14	0.0642	34.8
	0.11	11	0.0691	19.37	11	0.0653	27.49
0.07	0.08	170	0.0728	287.72	143	0.0713	343.95
	0.09	51	0.075	87.35	44	0.0722	107.39
	0.10	27	0.0769	46.61	24	0.0729	59.31
	0.11	18	0.0785	31.31	16	0.0733	39.95
	0.12	13	0.0792	22.84	12	0.0734	30.28
0.08	0.09	221	0.0829	372.66	182	0.0813	437.66
	0.10	64	0.0852	109.16	55	0.0823	133.81
	0.11	33	0.0871	56.8	29	0.083	71.35
	0.12	22	0.0891	38.05	19	0.0833	47.32
	0.13	16	0.0903	27.91	15	0.085	37.31
0.09	0.10	273	0.0929	460.3	230	0.0914	550.41
	0.11	79	0.0953	134.54	67	0.0923	162.95
	0.12	40	0.0973	68.69	35	0.0932	85.76
	0.13	26	0.0992	44.91	23	0.0939	56.86
	0.14	20	0.102	34.48	17	0.0945	42.34

**Table 2:** The parameters of the proposed plan for risks, $\alpha$ = 0.1 and $\beta$ = 0.05.

$CV_{AQL}$	$CV_{LTPD}$	*n*	*m*=2		*n*	*m*=3	
			*k*	*ASN*		*k*	*ASN*
0.05	0.06	99	0.0516	177.72	85	0.0502	221.65
	0.07	32	0.0526	58.14	28	0.05	74.19
	0.08	18	0.0533	32.99	17	0.0505	45.28
	0.09	13	0.0544	23.92	12	0.0504	32.25
	0.10	10	0.0547	18.52	10	0.0516	26.9
0.06	0.07	138	0.0617	246.93	116	0.0602	301.95
	0.08	43	0.0629	77.73	37	0.0601	97.62
	0.09	23	0.0635	42	21	0.0602	55.82
	0.10	16	0.0645	29.35	15	0.0607	40.04
	0.11	13	0.0665	23.83	12	0.0615	32.12
0.07	0.08	181	0.0717	323.74	155	0.0703	401.78
	0.09	55	0.073	99.19	48	0.0703	126.07
	0.10	29	0.0738	52.77	26	0.0702	68.92
	0.11	20	0.0751	36.51	18	0.0705	47.95
	0.12	15	0.0759	27.51	14	0.071	37.43
0.08	0.09	229	0.0817	409.34	197	0.0803	510.2
	0.10	68	0.083	122.53	59	0.0803	154.52
	0.11	36	0.0842	65.21	32	0.0805	84.34
	0.12	24	0.0853	43.69	21	0.0801	55.91
	0.13	18	0.0864	32.89	16	0.0804	42.76
0.09	0.10	290	0.0918	516.87	243	0.0903	628.45
	0.11	84	0.0931	151.16	72	0.0903	188.44
	0.12	43	0.0942	77.83	38	0.0904	100.07
	0.13	28	0.0952	50.93	25	0.0904	66.25
	0.14	21	0.0966	38.27	19	0.091	50.46

**Table 3:** The parameters of the proposed plan for risks, $\alpha$ = 0.05 and $\beta$ = 0.05.

$CV_{AQL}$	$CV_{LTPD}$	*n*	*m*=2		*n*	*m*=3	
			*k*	*ASN*		*k*	*ASN*
0.05	0.06	121	0.0524	212.46	103	0.0511	260.92
	0.07	39	0.0543	69.3	33	0.0516	85.39
	0.08	21	0.0553	37.9	19	0.0521	49.82
	0.09	15	0.057	27.18	14	0.0533	36.84
	0.10	12	0.0588	21.81	11	0.0539	29.17
0.06	0.07	169	0.0625	295.53	141	0.0611	356.71
	0.08	51	0.0643	90.52	45	0.0619	115.63
	0.09	28	0.066	50.11	25	0.0627	64.8
	0.10	19	0.0675	34.23	17	0.0631	44.57
	0.11	14	0.0681	25.47	13	0.0634	34.41
0.07	0.08	211	0.0725	386.28	188	0.0712	472.87
	0.09	66	0.0745	116.64	57	0.0719	146.09
	0.10	35	0.0762	62.41	31	0.0727	80.14
	0.11	23	0.0775	41.37	21	0.0735	54.65
	0.12	17	0.0786	30.79	16	0.0742	41.95
0.08	0.09	283	0.0825	494.78	239	0.0812	600.67
	0.10	83	0.0846	146.4	71	0.082	181.19
	0.11	43	0.0864	76.43	37	0.0825	95.63
	0.12	28	0.0879	50.15	25	0.0835	64.86
	0.13	21	0.0897	37.74	18	0.0833	47.29
0.09	0.10	356	0.0926	619.68	296	0.0912	743.31
	0.11	102	0.0947	179.53	86	0.092	219.09
	0.12	52	0.0966	92.15	45	0.0928	115.62
	0.13	33	0.098	58.98	29	0.0932	75.28
	0.14	24	0.0994	43.15	21	0.0934	54.98

**Table 4:** The parameters of the proposed plan for risks, $\alpha$ = 0.1 and $\beta$ = 0.1.

$CV_{AQL}$	$CV_{LTPD}$	*n*	*m*=2		*n*	*m*=3	
			*k*	*ASN*		*k*	*ASN*
0.05	0.06	75	0.0518	130.85	64	0.0502	160.25
	0.07	25	0.0532	43.96	22	0.0502	55.83
	0.08	14	0.0539	24.88	13	0.0503	33.25
	0.09	10	0.0548	17.87	10	0.0515	25.58
	0.10	8	0.0557	14.36	8	0.0518	20.61
0.06	0.07	103	0.0619	179.02	87	0.0602	217.49
	0.08	32	0.0631	56.27	28	0.06	71.03
	0.09	18	0.0643	31.83	16	0.06	40.87
	0.10	13	0.066	23.02	12	0.0612	30.6
	0.11	10	0.0669	17.81	9	0.0602	23.28
0.07	0.08	135	0.0719	234.63	116	0.0703	288.83
	0.09	42	0.0735	73.41	36	0.0702	90.86
	0.10	22	0.0743	38.85	20	0.0703	50.78
	0.11	15	0.0753	26.63	14	0.0706	35.73
	0.12	12	0.0774	21.27	11	0.0712	28.15
0.08	0.09	175	0.082	303.3	146	0.0803	362.86
	0.10	52	0.0835	90.88	44	0.0802	110.71
	0.11	27	0.0845	47.58	24	0.0802	60.87
	0.12	18	0.0856	31.86	17	0.0811	43.11
	0.13	14	0.0874	24.79	13	0.0815	33.11
0.09	0.10	216	0.092	374.25	181	0.0903	449.68
	0.11	63	0.0935	110.08	54	0.0903	135.59
	0.12	33	0.0949	57.93	29	0.0905	73.17
	0.13	21	0.0956	37.14	19	0.0902	48.36
	0.14	16	0.0972	28.33	15	0.0915	38.09

From the results in Tables 1, 2, 3, 4, we can observe that for fixed m, $\alpha$-risk and $\beta$-risk, the required sample size becomes smaller when the difference between the values of ${{\text{L}}}_{{\text{AQL}}}$ $CV_{AQL}$ and ${{\text{L}}}_{{\text{LTPD}}}$ $CV_{LTPD}$ becomes larger. This reason can be explained that it will be easier for us to make the correct judgment of lots because of a larger difference in agreed quality levels. Also, the required sample size would have an increasing trend while the stipulated risks become smaller. This phenomenon can be explained that if we expect that the chance of wrongly accepting bad lots or rejecting good lots is smaller, then we need to get more sample information to judge the lots. Furthermore, it can be seen that the required sample size becomes smaller and the corresponding critical acceptance value becomes smaller as the number of m increases. That is, the vendor can get the reduction of the required sample size with stricter acceptance standard as the resubmitted sampling plan provides a more flexible decision rule.

## Discussion and analysis

**Figure 1:**
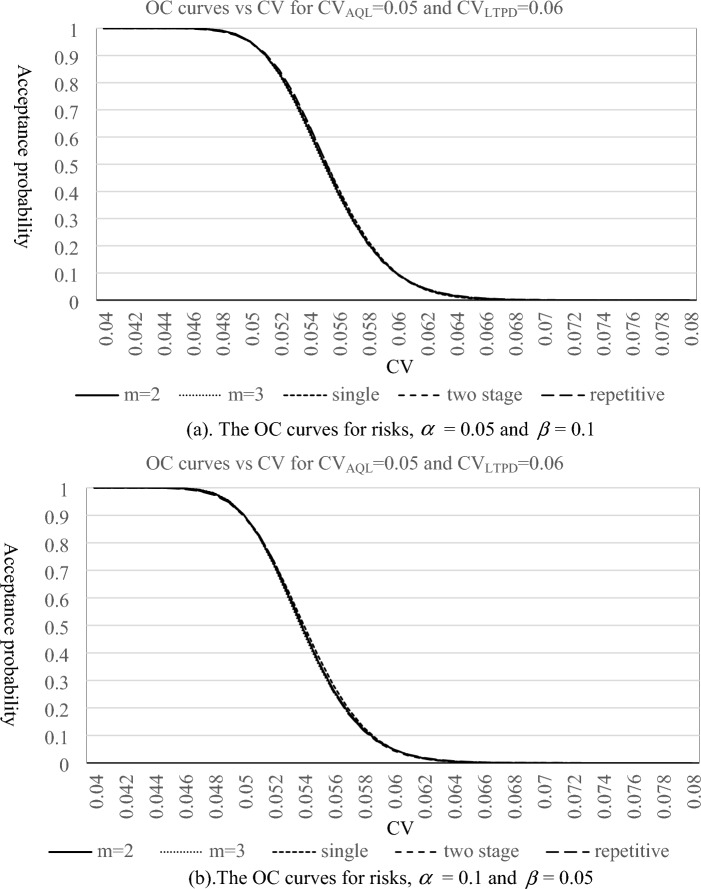
The OC curves for three plans with ${{\text{L}}}_{{\text{AQL}}}$ $CV_{AQL} = {0}{\text{.05}}$ and ${{\text{L}}}_{{\text{LTPD}}}$ $CV_{LTPD} = {0}{\text{.06}}$. *Note m* = 2, *m* = 3 means the proposed method. Single means single sampling plan based on *CV* (proposed by Tong and Chen^34^). Two stage means two stage sampling plan based on CV (proposed by Yan et al.^35^). Repetitive means repetitive group sampling plan based on CV (proposed by Yan et al.^36^).

**Figure 2:**
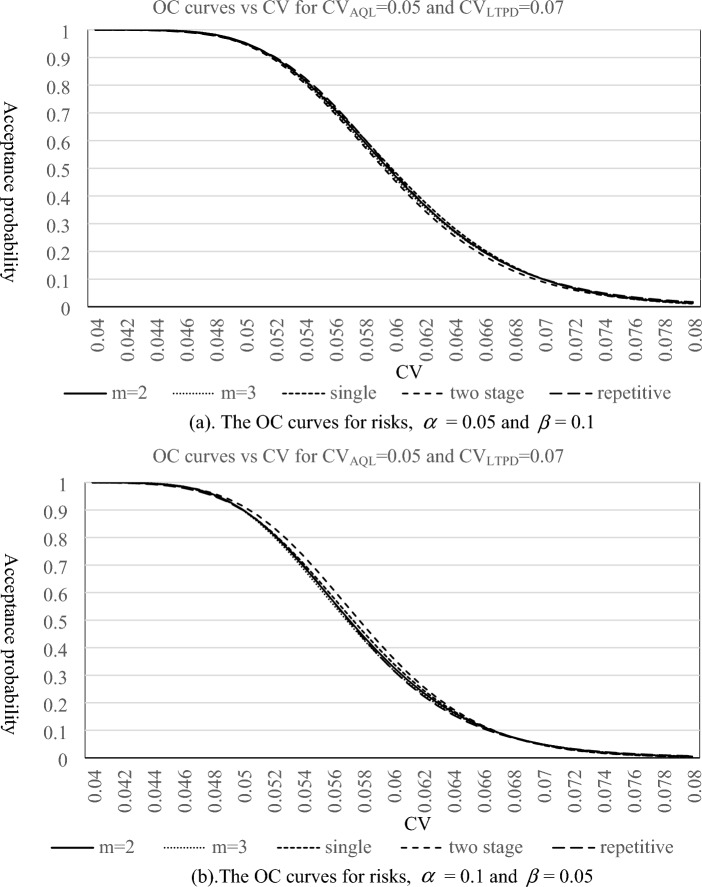
The OC curves for three plans with ${{\text{L}}}_{{\text{AQL}}}$ $CV_{AQL} = {0}{\text{.05}}$ and ${{\text{L}}}_{{\text{LTPD}}}$ $CV_{LTPD} = {0}{\text{.07}}$. *Note m* = 2, *m* = 3 means the proposed method. Single means single sampling plan based on *CV* (proposed by Tong and Chen^34^). Two stage means two stage sampling plan based on CV (proposed by Yan et al.^35^). Repetitive means repetitive group sampling plan based on CV (proposed by Yan et al.^36^).

In this section, we investigate the behaviors of the proposed plan and make a comparison with the existing CV-based sampling plans through OC curve and *ASN* curve. The OC curves of the proposed sampling plan for *m* = 2, 3, the CV-based single sampling plan^34^, two stage sampling plan^35^ and repetitive group sampling plan^36^ are depicted in Figs. 1 and 2, which consider the combinations of risks and quality levels for (${{\text{L}}}_{{\text{AQL}}}$ $CV_{AQL}$,${{\text{L}}}_{{\text{LTPD}}}$ $CV_{LTPD}$) = (0.05, 0.06) with ($\alpha$,$\beta$) = (0.05, 0.1) and (0.1, 0.05), and (${{\text{L}}}_{{\text{AQL}}}$ $CV_{AQL}$,${{\text{L}}}_{{\text{LTPD}}}$ $CV_{LTPD}$) = (0.05, 0.07) with ($\alpha$,$\beta$) = (0.05, 0.1) and (0.1, 0.05), respectively. From these graphs, we can see that the shapes of OC curves are very similar, which indicates that all sampling plans seem to have almost the same discriminatory power for lots. Nevertheless, we still can use the corresponding acceptance probabilities of one lot under various quality levels to compare the performance of these sampling plans. Tables 5, 6, 7, 8 display the corresponding acceptance probabilities of one lot under various quality levels for different CV-based sampling plans. For one lot with good quality levels (CV is below CV_AQL_), the proposed plan performs the best, followed by a single sampling plan and two stage sampling plan, and the repetitive sampling plan is the least. On the other hand, for one lot with bad quality levels (CV is above CV_LTPD_), two stage sampling plan performs the best, followed by a single sampling plan and the proposed plan, and the repetitive sampling plan is the least. Therefore, the proposed plan and two stage sampling plan can generally be thought of as having better performances on the discrimination of lot quality than the other two sampling plans.

**Table 5:** The corresponding acceptance probabilities of lot under various quality levels for (CV_AQL_, CV_LTPD_) = (0.05, 0.06) with ($\alpha$,$\beta$) = (0.05, 0.1).

CV	m = 2	m = 3	Single	Two stage	Repetitive
**0.04**	**1**	**1**	**0.999999996**	**0.999999958**	**0.999999787**
**0.041**	**0.999999994**	**0.999999998**	**0.999999939**	**0.99999961**	**0.999998431**
**0.042**	**0.999999875**	**0.999999956**	**0.999999285**	**0.99999718**	**0.999990773**
**0.043**	**0.999998356**	**0.999999242**	**0.999993766**	**0.999983578**	**0.999955549**
**0.044**	**0.999984626**	**0.999991204**	**0.999958478**	**0.999920901**	**0.999821019**
**0.045**	**0.999894112**	**0.999928123**	**0.999782791**	**0.999676813**	**0.999384848**
**0.046**	**0.999444733**	**0.999569774**	**0.999085389**	**0.998857116**	**0.998161995**
**0.047**	**0.997716225**	**0.998045348**	**0.996830297**	**0.996455958**	**0.995142609**
**0.048**	**0.99243195**	**0.993037693**	**0.990776363**	**0.990298937**	**0.988469192**
**0.049**	**0.979291639**	**0.979975558**	**0.97705089**	**0.97648985**	**0.975093337**
**0.05**	**0.952147997**	**0.952222356**	**0.95036389**	**0.949425239**	**0.950576483**
0.051	0.904667828	0.90305597	0.905244965	0.903011323	0.909378891
0.052	0.833090394	0.828859525	0.838083938	0.833077206	0.846141755
0.053	0.738581337	0.731585376	0.74906859	0.739895211	0.758226226
0.054	0.627650359	0.618778752	0.642942191	0.629206466	0.648642943
0.055	0.510311978	0.501146049	0.528074217	0.510906769	0.527023848
0.056	0.397055148	0.389193645	0.414252671	0.396033211	0.406751135
0.057	0.296159767	0.290649262	0.310212799	0.293655332	0.299705346
0.058	0.212343835	0.209472179	0.221879317	0.209050025	0.212497222
0.059	0.146817329	0.146247397	0.151777135	0.143550461	0.146185965
0.06	0.098215353	0.099279019	0.099476218	0.09556933	0.098268382
**0.061**	**0.063773534**	**0.065751402**	**0.062601472**	**0.061994359**	**0.064884143**
**0.062**	**0.040313719**	**0.042612062**	**0.037914637**	**0.039356706**	**0.042226337**
**0.063**	**0.024876087**	**0.027093664**	**0.022152488**	**0.024539568**	**0.027143593**
**0.064**	**0.015019802**	**0.016938585**	**0.012516082**	**0.015067628**	**0.017255006**
**0.065**	**0.008892334**	**0.010432481**	**0.006854278**	**0.009127191**	**0.010854648**
**0.066**	**0.005171881**	**0.006340302**	**0.003646585**	**0.005460691**	**0.006759856**
**0.067**	**0.002959961**	**0.003807681**	**0.001888793**	**0.003229226**	**0.004168724**
**0.068**	**0.001669467**	**0.002262457**	**0.000954446**	**0.0018885**	**0.002546451**
**0.069**	**0.000929207**	**0.001331524**	**0.000471449**	**0.001092698**	**0.001541269**
**0.07**	**0.000511007**	**0.000776957**	**0.00022805**	**0.000625823**	**0.000924704**
**0.071**	**0.000277979**	**0.000449898**	**0.000108215**	**0.000354974**	**0.000550204**
**0.072**	**0.000149735**	**0.000258734**	**5.04559E-05**	**0.000199521**	**0.00032483**
**0.073**	**7.99423E−05**	**0.000147892**	**2.32E−05**	**0.000111198**	**0.00019039**
**0.074**	**4.23E−05**	**8.41E−05**	**1.05E−05**	**6.15E−05**	**0.000110851**
**0.075**	**2.23E−05**	**4.76E−05**	**4.67E−06**	**3.38E−05**	**6.415E−05**
**0.076**	**1.16E−05**	**2.68E−05**	**2.06E−06**	**1.84E−05**	**3.69199E−05**
**0.077**	**6.04E−06**	**1.50E−05**	**8.97E−07**	**9.99E−06**	**2.11432E−05**
**0.078**	**3.12E−06**	**8.42E−06**	**3.87E−07**	**5.39E−06**	**1.20549E−05**
**0.079**	**1.61E−06**	**4.70E−06**	**1.66E−07**	**2.89E−06**	**6.85E−06**
**0.08**	**8.25E−07**	**2.62E−06**	**7.03E−08**	**1.55E−06**	**3.87E−06**

Significance values are in bold.

*m* = 2, *m* = 3 means the proposed method.

Single means single sampling plan based on *CV* (proposed by Tong and Chen^34^).

Two stage means two stage sampling plan based on CV (proposed by Yan et al.^35^).

Repetitive means repetitive group sampling plan based on CV (proposed by Yan et al.^36^).

**Table 6:** The corresponding acceptance probabilities of lot under various quality levels for (CV_AQL_, CV_LTPD_) = (0.05, 0.06) with ($\alpha$,$\beta$) = (0.1, 0.05).

CV	m = 2	m = 3	Single	Two stage	Repetitive
**0.04**	**1**	**1**	**1**	**1**	**0.999999**
**0.041**	**1**	**1**	**1**	**0.999997**	**0.999994**
**0.042**	**0.999999**	**1**	**0.999996**	**0.999981**	**0.999967**
**0.043**	**0.999991**	**0.999996**	**0.99997**	**0.999908**	**0.999856**
**0.044**	**0.999931**	**0.999963**	**0.999828**	**0.999628**	**0.999467**
**0.045**	**0.999591**	**0.999731**	**0.999219**	**0.998712**	**0.998309**
**0.046**	**0.998135**	**0.998583**	**0.997123**	**0.996093**	**0.99531**
**0.047**	**0.993297**	**0.994324**	**0.991215**	**0.989518**	**0.988427**
**0.048**	**0.980491**	**0.982145**	**0.977326**	**0.974983**	**0.974216**
**0.049**	**0.952825**	**0.954495**	**0.949621**	**0.946709**	**0.947586**
0.05	0.902995	0.90329	0.902027	0.898326	0.902182
0.051	0.826679	0.824023	0.830649	0.825367	0.83208
0.052	0.725492	0.719105	0.736044	0.728015	0.735077
0.053	0.607393	0.597879	0.623998	0.612376	0.616144
0.054	0.4842	0.47328	0.504229	0.4892	0.487765
0.055	0.367733	0.357396	0.387633	0.370586	0.365343
0.056	0.26667	0.258379	0.283406	0.266441	0.260748
0.057	0.185253	0.179604	0.197215	0.182328	0.179004
0.058	0.123742	0.120578	0.130831	0.119274	0.119271
0.059	0.079776	0.078516	0.082918	0.075001	0.077679
0.06	0.049817	0.049777	0.050328	0.045615	0.049693
**0.061**	**0.030231**	**0.030825**	**0.02933**	**0.027006**	**0.031318**
**0.062**	**0.01788**	**0.018699**	**0.016456**	**0.01566**	**0.019479**
**0.063**	**0.010332**	**0.011138**	**0.008912**	**0.008943**	**0.011968**
**0.064**	**0.005847**	**0.006528**	**0.004671**	**0.005052**	**0.007267**
**0.065**	**0.003247**	**0.003771**	**0.002375**	**0.002832**	**0.004362**
**0.066**	**0.001773**	**0.00215**	**0.001174**	**0.001578**	**0.00259**
**0.067**	**0.000953**	**0.001212**	**0.000566**	**0.000875**	**0.001521**
**0.068**	**0.000505**	**0.000676**	**0.000266**	**0.000483**	**0.000884**
**0.069**	**0.000264**	**0.000374**	**0.000123**	**0.000265**	**0.000509**
**0.07**	**0.000137**	**0.000205**	**5.54E-05**	**0.000145**	**0.00029**
**0.071**	**7E−05**	**0.000111**	**2.46E−05**	**7.88E−05**	**0.000164**
**0.072**	**3.55E−05**	**6.02E−05**	**1.07E−05**	**4.26E−05**	**9.23E−05**
**0.073**	**1.78E−05**	**3.23E−05**	**4.60E−06**	**2.29E−05**	**5.14E−05**
**0.074**	**8.91E−06**	**1.73E−05**	**1.95E−06**	**1.22E−05**	**2.85E−05**
**0.075**	**4.42E−06**	**9.19E−06**	**8.16E−07**	**6.52E−06**	**1.57E−05**
**0.076**	**2.18E−06**	**4.87E−06**	**3.38E−07**	**3.46E−06**	**8.60E−06**
**0.077**	**1.07E−06**	**2.57E−06**	**1.39E−07**	**1.82E−06**	**4.69E−06**
**0.078**	**5.21E−07**	**1.36E−06**	**5.63E−08**	**9.60E−07**	**2.55E−06**
**0.079**	**2.54E−07**	**7.13E−07**	**2.27E−08**	**5.03E−07**	**1.38E−06**
**0.08**	**1.23E−07**	**3.74E−07**	**9.08E−09**	**2.63E−07**	**7.45E−07**

Significance values are in bold.

*m* = 2, *m* = 3 means the proposed method.

Single means single sampling plan based on *CV* (proposed by Tong and Chen^34^).

Two stage means two stage sampling plan based on CV (proposed by Yan et al.^35^).

Repetitive means repetitive group sampling plan based on CV (proposed by Yan et al.^36^).

**Table 7:** The corresponding acceptance probabilities of lot under various quality levels for (CV_AQL_, CV_LTPD_) = (0.05, 0.07) with ($\alpha$,$\beta$) = (0.05, 0.1).

CV	m = 2	m = 3	Single	Two stage	Repetitive
**0.04**	**0.999990364**	**0.999994368**	**0.999969476**	**0.999953095**	**0.999874161**
**0.041**	**0.999962599**	**0.999975512**	**0.9999034**	**0.999869384**	**0.9996999**
**0.042**	**0.999874745**	**0.999909815**	**0.999728406**	**0.999666966**	**0.999339262**
**0.043**	**0.999632363**	**0.999713566**	**0.99931335**	**0.999215883**	**0.998644956**
**0.044**	**0.999041123**	**0.999202798**	**0.99842211**	**0.998283902**	**0.997391132**
**0.045**	**0.997750141**	**0.998027881**	**0.996672817**	**0.996490572**	**0.995251785**
**0.046**	**0.995198831**	**0.995608546**	**0.993507423**	**0.993265212**	**0.991781342**
**0.047**	**0.990590197**	**0.99109791**	**0.988186269**	**0.987825842**	**0.986401189**
**0.048**	**0.982913441**	**0.983405504**	**0.979819484**	**0.979197731**	**0.978397176**
**0.049**	**0.971027976**	**0.97129557**	**0.967438967**	**0.966284101**	**0.96693456**
0.05	0.953802305	0.953551838	0.95010388	0.947988066	0.951097638
0.051	0.93028224	0.929174751	0.927022634	0.923368278	0.929960557
0.052	0.899851587	0.897563398	0.897668842	0.891797549	0.902691616
0.053	0.862349119	0.858637857	0.861869271	0.8530895	0.868684964
0.054	0.81811747	0.812875637	0.819848003	0.807564732	0.827701302
0.055	0.767977227	0.761259985	0.772220476	0.756042715	0.779986749
0.056	0.713136753	0.705158819	0.719941035	0.699763114	0.726333022
0.057	0.655059989	0.646165112	0.664215342	0.640254986	0.668049276
0.058	0.595318815	0.585931264	0.606393388	0.579180547	0.606838058
0.059	0.535454111	0.52602385	0.547859364	0.51818085	0.544597793
0.06	0.476862812	0.467814926	0.489932202	0.458745499	0.4831982
0.061	0.420719997	0.412415627	0.43378642	0.402119796	0.424281276
0.062	0.367937366	0.360649541	0.380398086	0.349253673	0.369126869
0.063	0.319153921	0.313058207	0.330516462	0.300789276	0.318597101
0.064	0.274751503	0.269929061	0.284658598	0.257079232	0.27315052
0.065	0.234886866	0.231336261	0.243122198	0.21822568	0.232903171
0.066	0.199532522	0.197186573	0.20601125	0.18413009	0.197711336
0.067	0.168520023	0.167264377	0.173269063	0.154545929	0.167255729
0.068	0.141581207	0.141272174	0.144714092	0.129128023	0.14111475
0.069	0.11838454	0.118864635	0.120074964	0.107475125	0.118821534
0.07	0.098565207	0.099675597	0.099022252	0.089163989	0.099904393
**0.071**	**0.081748632**	**0.083338303**	**0.081195573**	**0.073774789**	**0.083912919**
**0.072**	**0.067567795**	**0.069499664**	**0.066225403**	**0.060908636**	**0.070432968**
**0.073**	**0.05567516**	**0.057829564**	**0.053749643**	**0.050198457**	**0.05909368**
**0.074**	**0.045750132**	**0.048026261**	**0.043425373**	**0.041314679**	**0.049569218**
**0.075**	**0.037503049**	**0.039818832**	**0.034936469**	**0.033967079**	**0.041577252**
**0.076**	**0.030676567**	**0.032967532**	**0.027997848**	**0.027904006**	**0.034875633**
**0.077**	**0.025045219**	**0.027262708**	**0.022357106**	**0.022909947**	**0.029258228**
**0.078**	**0.020413759**	**0.022522831**	**0.017794239**	**0.018802165**	**0.024550521**
**0.079**	**0.016614777**	**0.018592013**	**0.014120062**	**0.015426948**	**0.020605349**
**0.08**	**0.013505935**	**0.015337326**	**0.011173794**	**0.012655817**	**0.017298975**

Significance values are in bold.

*m* = 2, *m* = 3 means the proposed method.

Single means single sampling plan based on *CV* (proposed by Tong and Chen^34^).

Two stage means two stage sampling plan based on CV (proposed by Yan et al.^35^).

Repetitive means repetitive group sampling plan based on CV (proposed by Yan et al.^36^).

**Table 8:** The corresponding acceptance probabilities of lot under various quality levels for (CV_AQL_, CV_LTPD_) = (0.05, 0.07) with ($\alpha$,$\beta$) = (0.1, 0.05).

CV	m = 2	m = 3	Single	Two stage	Repetitive
**0.04**	**0.999948217**	**0.999967777**	**0.999880621**	**0.999809192**	**0.999627566**
**0.041**	**0.999820967**	**0.999875597**	**0.999651749**	**0.999531109**	**0.999148281**
**0.042**	**0.99946299**	**0.999591317**	**0.999094633**	**0.998936711**	**0.998199532**
**0.043**	**0.9985809**	**0.998836733**	**0.997877127**	**0.997757064**	**0.99644997**
**0.044**	**0.996650787**	**0.997084901**	**0.995462705**	**0.995570033**	**0.9934194**
**0.045**	**0.992854513**	**0.993476539**	**0.991076467**	**0.991764053**	**0.988453754**
**0.046**	**0.986069449**	**0.986797813**	**0.983714843**	**0.985522942**	**0.980713513**
**0.047**	**0.974942267**	**0.975562436**	**0.972214133**	**0.975847706**	**0.969185347**
**0.048**	**0.958052516**	**0.958207711**	**0.955377181**	**0.96162626**	**0.952728775**
**0.049**	**0.934138564**	**0.933367726**	**0.93213902**	**0.941750859**	**0.930169327**
0.05	0.90233251	0.900151556	0.90173847	0.915268747	0.90044412
0.051	0.862344001	0.8583481	0.863858799	0.881539172	0.862791956
0.052	0.814547687	0.80850374	0.818707619	0.840364412	0.816958627
0.053	0.759957616	0.751860451	0.767021185	0.792066066	0.763365604
0.054	0.700102113	0.690181578	0.709995669	0.737489684	0.703180269
0.055	0.636834192	0.625516212	0.649162531	0.67793673	0.638242
0.056	0.572120546	0.559957048	0.586233476	0.615038013	0.570842234
0.057	0.507847724	0.495435122	0.522941825	0.550593022	0.50341037
0.058	0.44567189	0.433575974	0.460902786	0.486402998	0.43819178
0.059	0.386923988	0.375623216	0.401507476	0.424122622	0.377000264
0.06	0.332569561	0.322421747	0.345857084	0.365147735	0.321090376
0.061	0.283213936	0.274445488	0.29473626	0.31054733	0.271148951
0.062	0.239139553	0.23185264	0.248619677	0.261039561	0.227372534
0.063	0.200361834	0.194553344	0.207702987	0.217005051	0.189587314
0.064	0.166691984	0.162278328	0.17194882	0.178527386	0.157374807
0.065	0.137798189	0.134641065	0.141139373	0.145449642	0.130180779
0.066	0.113259794	0.111189703	0.114928899	0.117436896	0.107398081
0.067	0.092611794	0.091447424	0.092891475	0.094036845	0.088423113
0.068	0.075378961	0.074941792	0.074561352	0.074733246	0.072690173
0.069	0.061100254	0.06122455	0.059464788	0.058989357	0.059689381
0.07	0.049344881	0.049883671	0.047143468	0.046280541	0.048973404
**0.071**	**0.03972164**	**0.040549519**	**0.037170305**	**0.0361166**	**0.040157245**
**0.072**	**0.031883193**	**0.032896787**	**0.02915889**	**0.028055256**	**0.032914097**
**0.073**	**0.025526703**	**0.026643556**	**0.022767921**	**0.021708573**	**0.026969312**
**0.074**	**0.020392056**	**0.021548569**	**0.017701932**	**0.016744146**	**0.022093749**
**0.075**	**0.016258612**	**0.017407503**	**0.013709468**	**0.012882718**	**0.018097243**
**0.076**	**0.012941176**	**0.014048817**	**0.010579658**	**0.009893604**	**0.014822577**
**0.077**	**0.010285688**	**0.011329531**	**0.008137916**	**0.007588987**	**0.012140128**
**0.078**	**0.008164976**	**0.009131207**	**0.006241318**	**0.005817862**	**0.009943243**
**0.079**	**0.006474746**	**0.007356244**	**0.004774015**	**0.004460132**	**0.008144286**
**0.08**	**0.005129959**	**0.005924568**	**0.00364293**	**0.003421175**	**0.006671325**

Significance values are in bold.

*m* = 2, *m* = 3 means the proposed method.

Single means single sampling plan based on *CV* (proposed by Tong and Chen^34^).

Two stage means two stage sampling plan based on CV (proposed by Yan et al.^35^).

Repetitive means repetitive group sampling plan based on CV (proposed by Yan et al.^36^).

**Figure 3:**
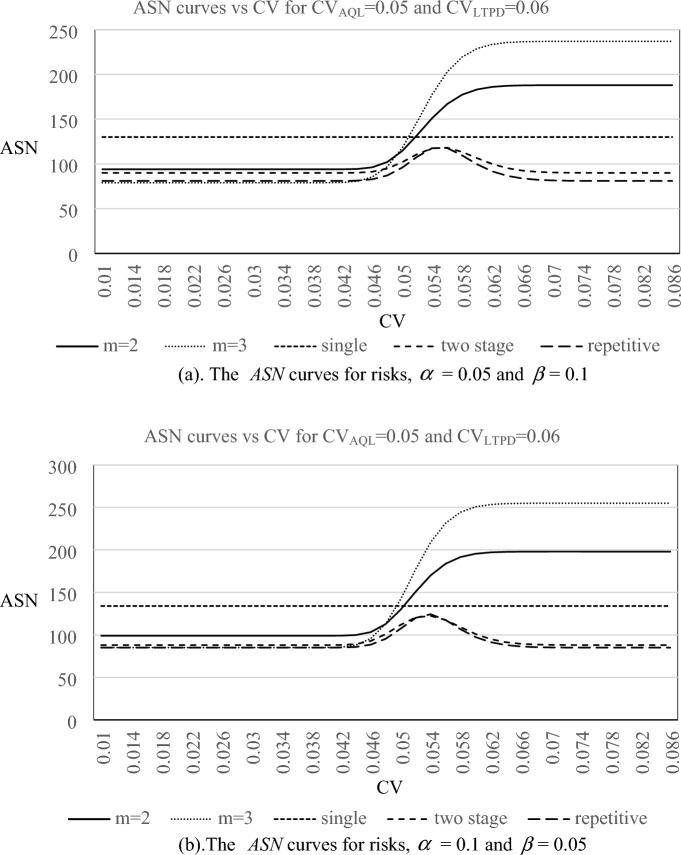
The *ASN* curves for three plans with ${{\text{L}}}_{{\text{AQL}}}$ $CV_{AQL} = {0}{\text{.05}}$ and ${{\text{L}}}_{{\text{LTPD}}}$ $CV_{LTPD} = {0}{\text{.06}}$. *Note m* = 2, *m* = 3 means the proposed method. Single means single sampling plan based on *CV* (proposed by Tong and Chen^34^). Two stage means two stage sampling plan based on CV (proposed by Yan et al.^35^). Repetitive means repetitive group sampling plan based on CV (proposed by Yan et al.^36^).

**Figure 4:**
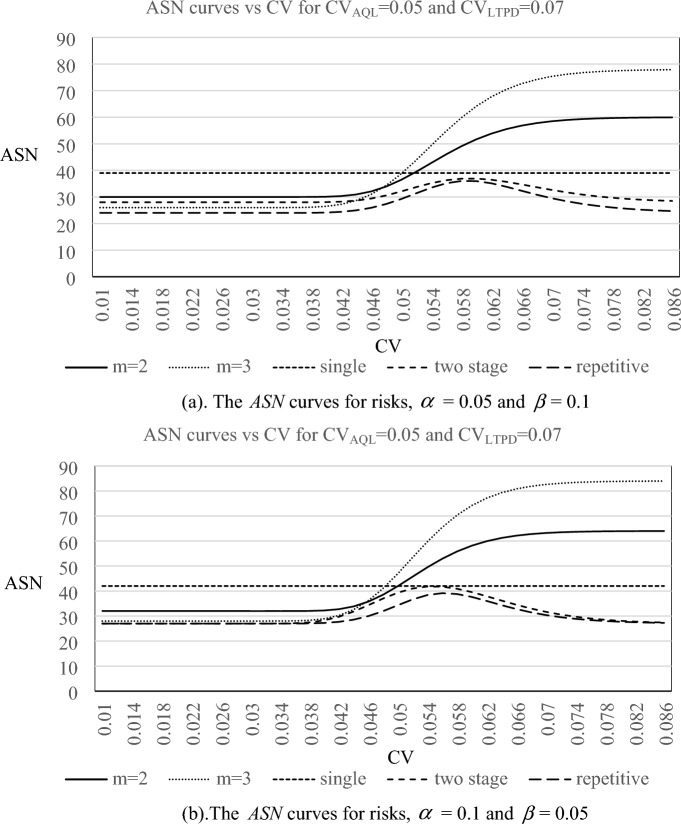
The *ASN* curves for three plans with ${{\text{L}}}_{{\text{AQL}}}$ $CV_{AQL} = {0}{\text{.05}}$ and ${{\text{L}}}_{{\text{LTPD}}}$ $CV_{LTPD} = {0}{\text{.07}}$. *Note m* = 2, *m* = 3 means the proposed method. Single means single sampling plan based on *CV* (proposed by Tong and Chen^34^). Two stage means two stage sampling plan based on CV (proposed by Yan et al.^35^). Repetitive means repetitive group sampling plan based on CV (proposed by Yan et al.^36^).

In addition, the ASN curves of the proposed resubmitted sampling plan, CV-based single sampling plan^34^, two stage sampling plan^35^ and repetitive group sampling plan^36^ for quality levels (CV_AQL_, CV_LTPD_) = (0.05, 0.06), and (0.05,0.07) are plotted in Figs. 3 and 4, respectively. From the appearances of ASN curves, we can observe the following results:

1. The ASN curves have the same trend for all combinations of risks and quality levels.
2. The resubmitted sampling plan would need more sample size as the value of CV becomes larger while the resubmitted sampling just needs less sample size as the value of CV decreases. This phenomenon is reasonable because those lots judged as not acceptable have to be resampled even if the original inspection displayed evidence of poor quality.
3. For lots with good quality levels, the resubmitted sampling plan with m = 3 needs less sample size to reach lot sentencing than that of the resubmitted sampling plan with m = 2. On the other hand, for lots with bad quality levels, the resubmitted sampling plan with m = 2 needs less sample size to reach lot sentencing than that of the resubmitted sampling plan with m = 3.
4. For lots with good quality levels and bad quality levels, two stage and repetitive sampling plans just need less sample size to reach lot sentencing. Instead, when one lot with general quality level (about between CV_AQL_ and CV_LTPD_), two stage and repetitive sampling plans need a larger sample size to reach lot sentencing.

## An application example in industry

To illustrate how the proposed methodology can be applied to the actual situation of lot inspection, one example of a milk bottling plant is presented. In milk bottling plants, there are several sizes of containers for the installation of milk. For the wholesalers who receive the goods shipped from the milk bottling plants, the volume of milk in containers is a critical characteristic. Owing to their long-term purchasing, they may be more concerned with the stability of volume for one batch of milk than the average volume for one batch of milk since the users who drink usually care about the consistency of products. Because of the different sizes of containers, the volumes of milk usually have a standard deviation proportional to the mean. To build the united criteria for various containers, the *CV* index can be regarded as a suitable tool for the evaluation of stability. More applications of sampling plans can be seen in^37–41^.

By virtue of the extended partnership between plants and wholesalers, the wholesalers are willing to adopt the resubmitted sampling plan which permits the resampling to be executed for lots non-accepted. Suppose a particular type of container fitting milk is investigated, and the specification limits of the volume are *LSL* = 290 mL and *USL* = 310 mL. In the contract approved by the two parties, the quality level of ${{\text{L}}}_{{\text{AQL}}}$ $CV_{AQL}$ and ${{\text{L}}}_{{\text{LTPD}}}$ $CV_{LTPD}$ are set to 0.05 and 0.07 with the $\alpha$ = 0.05 and $\beta$ = 0.10, and the times of resampling permitted is two (that is, *m* = 3). By looking up Table 1, we can find the parameters of the resubmitted sampling plan are (*n*, $k$) = (26, 0.0519), which implies that the lot will be accepted if 26 items yield measurements with $\mathop {CV}\limits^{ \wedge }$ < 0.0519, and the consumer will allow resubmissions twice in the case of non-acceptance on the submitted sample, and will reject the lot if two resubmissions with the same sample size is still not accepted.

**Figure 5:**
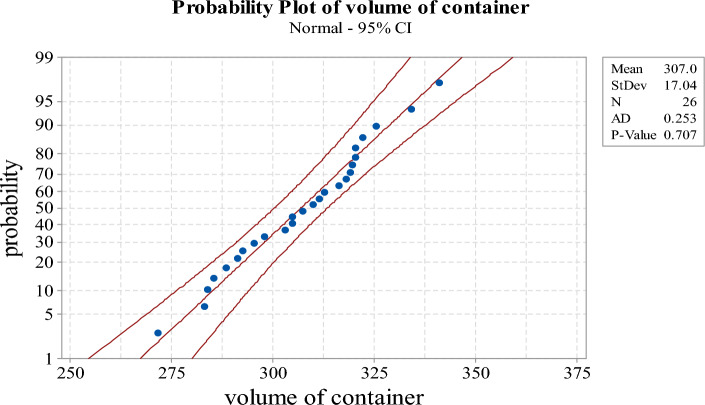
The normal probability plot for 26 containers fitting milk (original sample data).

Twenty-six observations are randomly taken from the lot as shown in Table 9. We depict the normal probability plot of sample data and use the *p*-value to test if the sample data obeys a normal distribution through Minitab software, which is shown in Fig. 5. The StDev, N and AD of the upper right side in the Figure represent the standard deviation, number of observations and Anderson–Darling statistic, respectively. From the graph and *p*-value, we can conclude that the sample data follows the normal distribution. Based on these observations, we can obtain

$$ \overline{X} = {306}{\text{.9677}},\;SA = {17}{\text{.03986}},\;\;{\text{and}}\;\;\mathop {CV}\limits^{ \wedge } = \frac{S}{{\overline{X}}} = \frac{{{17}{\text{.03986}}}}{{{306}{\text{.9677}}}} = {0}{\text{.05551}}. $$

**Table 9:** The volume of 26 containers fitting milk (original sample data).

311.54	319.57	295.31	318.01	285.47	304.87	304.87
292.70	288.51	307.45	291.41	320.35	283.85	334.09
316.35	320.38	322.30	303.05	312.72	297.85	271.63
319.02	325.49	341.20	283.25	309.92

**Figure 6:**
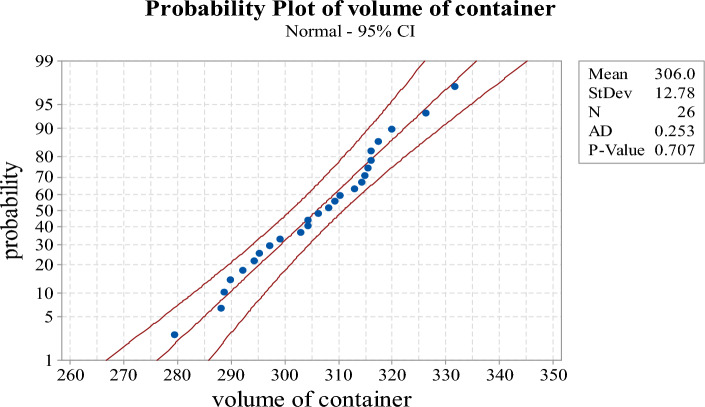
The normal probability plot for 26 containers fitting milk (resubmitted sample data).

Since the value of $\mathop {CV}\limits^{ \wedge }$ is larger than 0.0519, this lot is not accepted and a new sample of size 26 should be further inspected. The observed measurements of the resubmitted samples are shown in Table 10. Figure 6 depicts the normal probability plot of the new sample data. From the graph and *p*-value, it is concluded that the new sample data obey the normal distribution. The statistics from the resubmitted sample are

$$ \overline{X} = {305}{\text{.9762}},\;S = {12}{\text{.77983}},\;\;{\text{and}}\;\;\mathop {CV}\limits^{ \wedge } = \frac{S}{{\overline{X}}} = \frac{{{12}{\text{.77983}}}}{{{305}{\text{.9762}}}} = {0}{\text{.041767}}. $$

**Table 10:** The volume of 26 containers fitting milk (resubmitted sample data).

309.40	315.43	297.23	314.26	289.85	304.40	304.41
295.28	292.13	306.34	294.31	316.02	288.64	326.32
313.01	316.03	317.47	303.04	310.29	299.14	279.47
315.01	319.87	331.65	288.19	308.19

Since the value of $\mathop {CV}\limits^{ \wedge }$ is less than 0.0519, this lot would be accepted.

## Conclusions

The *CV* considers the degree of a standard deviation relative to the mean, which is used as a measure of variability for circumstances when the standard deviation is proportional to the mean or measurements are made in different units. In this paper, a new sampling plan based on the coefficient of variation for the resubmitted lot is developed. For the discriminatory power of lot, the proposed plan and the existing single sampling plan based on a *CV* are almost the same. However, the proposed plan would need less sample size when the quality of the submitted lot is good in comparison to that of the existing single sampling plan based on a *CV*. Therefore, the proposed resubmitted sampling plan could be recommended for lots with higher quality levels of *CV* as well as dealing with the demand for resubmitted lots. It is suggested that further research can be extended for the resubmitted sampling plan with non-normal distribution consideration.

## Data Availability

The data is available from Muhammad Aslam (aslam_ravian@hotmail.com).
